# Ascorbic Acid Mitigates D-galactose-Induced Brain Aging by Increasing Hippocampal Neurogenesis and Improving Memory Function

**DOI:** 10.3390/nu11010176

**Published:** 2019-01-15

**Authors:** Sung Min Nam, Misun Seo, Jin-Seok Seo, Hyewhon Rhim, Sang-Soep Nahm, Ik-Hyun Cho, Byung-Joon Chang, Hyeon-Joong Kim, Sun-Hye Choi, Seung-Yeol Nah

**Affiliations:** 1 Department of Anatomy, College of Veterinary Medicine, Konkuk University, Seoul 05029, Korea; skavet@konkuk.ac.kr (S.M.N.); phoenix_1st@naver.com (J.-S.S.); ssnahm@konkuk.ac.kr (S.-S.N.); bjchang@konkuk.ac.kr (B.-J.C.); 2 Center for Neuroscience, Korea Institute of Science and Technology, Seoul 02792, Korea; misun@kist.re.kr (M.S.); hrhim@kist.re.kr (H.R.); 3 Department of Science in Korean Medicine, Brain Korea 21 Plus Program, Kyung Hee University, Seoul 02447, Korea; ihcho@khu.ac.kr; 4 Department of Cancer Preventive Material Development, Graduate School, Kyung Hee University, Seoul 02447, Korea; 5 Ginsentology Research Laboratory and Department of Physiology, College of Veterinary Medicine, Konkuk University, Seoul 05029, Korea; hyunjoongk@gmail.com (H.-J.K.); vettman@konkuk.ac.kr (S.-H.C.)

**Keywords:** ascorbic acid, D-galactose, hippocampus, brain aging, neurogenesis

## Abstract

Ascorbic acid is essential for normal brain development and homeostasis. However, the effect of ascorbic acid on adult brain aging has not been determined. Long-term treatment with high levels of D-galactose (D-gal) induces brain aging by accumulated oxidative stress. In the present study, mice were subcutaneously administered with D-gal (150 mg/kg/day) for 10 weeks; from the seventh week, ascorbic acid (150 mg/kg/day) was orally co-administered for four weeks. Although D-gal administration alone reduced hippocampal neurogenesis and cognitive functions, co-treatment of ascorbic acid with D-gal effectively prevented D-gal-induced reduced hippocampal neurogenesis through improved cellular proliferation, neuronal differentiation, and neuronal maturation. Long-term D-gal treatment also reduced expression levels of synaptic plasticity-related markers, i.e., synaptophysin and phosphorylated Ca^2+^/calmodulin-dependent protein kinase II, while ascorbic acid prevented the reduction in the hippocampus. Furthermore, ascorbic acid ameliorated D-gal-induced downregulation of superoxide dismutase 1 and 2, sirtuin1, caveolin-1, and brain-derived neurotrophic factor and upregulation of interleukin 1 beta and tumor necrosis factor alpha in the hippocampus. Ascorbic acid-mediated hippocampal restoration from D-gal-induced impairment was associated with an enhanced hippocampus-dependent memory function. Therefore, ascorbic acid ameliorates D-gal-induced impairments through anti-oxidative and anti-inflammatory effects, and it could be an effective dietary supplement against adult brain aging.

## 1. Introduction

With an increased life expectancy, aging-related cognitive impairments have become an important social issue because of impaired quality of life in the elderly. In addition, brain aging is the main causing factor in the development of neurodegenerative diseases [1]. Accumulation of oxidative stress is one of the contributing mechanisms underlying accelerated brain aging, and antioxidants as preventive medicine have been attracting attention because they may delay brain aging [2,3,4]. D-galactose (D-gal), a monosaccharide, is commonly found in dairy products, avocado, sugar beet, and mucilage. D-gal is normally metabolized into galactose-1-phosphate, but at high levels, D-gal causes the accumulation of galactitol and subsequent formation of advanced glycation end-products [5]. Banji et al. (2013) also reported that D-gal (150 mg/kg) treatment caused oxidative damage by increasing protein carbonyls and advanced oxidation protein products in the rat brain [6]. Moreover, chronic systemic treatment with high levels of D-gal accelerates brain aging through accumulated oxidative stress [2,4,7,8,9]. As occurs during natural brain aging in humans, D-gal-induced brain senescence animals exhibit brain aging phenotypes, including memory impairment and adult hippocampal neurogenesis reduction [8,9,10].

Ascorbic acid (reduced form of vitamin C) is essential because it functions as an electron donor and a cofactor for enzyme activation and is involved in collagen synthesis, wound healing, bleeding prevention, antioxidation, and regulation of the immune response [11]. Therefore, supplementation of ascorbic acid is recommended. In addition, ascorbic acid can be acquired from natural sources, mainly from fruits and vegetables and in small quantities from meat and milk [12]. Rats, mice, dogs, and cats can synthesize ascorbic acid from glucose in the liver, while humans and guinea pigs must be supplemented with ascorbic acid from external sources [13]. The concentration of ascorbic acid is relatively higher in the brain than in other organs. Notably, the concentration of ascorbic acid is higher in the hippocampus, amygdala, cerebral cortex, and hypothalamus than in other brain areas [14]. Notably, the concentration of ascorbic acid peaks at the postnatal period, and subsequently, its level decreases with aging [15]. Moreover, the blood level of ascorbic acid has an inverse relationship with the mortality of chronic diseases, such as cardiovascular disease, ischemic heart disease, and cancer [16]. However, whether decreased levels of ascorbic acid are responsible for brain aging is an unsolved issue, and the effects of ascorbic acid supplementation on the hippocampal structure and hippocampus-dependent memory in the aged brain are unclear. Therefore, the present study investigated the effects of the oral administration of ascorbic acid on adult hippocampal neurogenesis and subsequent memory dysfunction in a D-gal-induced brain senescence animal model.

## 2. Materials and Methods

### 2.1. Animals

Male C57BL/6 mice aged five weeks were purchased from NaraBio (Narabio Co., Seoul, Republic of Korea). Animals were housed in a conventional state under adequate temperature (23 °C) and humidity (60%) control with a 12-h light/12-h dark cycle and were allowed free access to food (Purina 5008, Purina Korea, Korea) and tap water. The experimental protocols and procedures were approved by the Institutional Animal Care and Use Committee of the Konkuk University (Permit number 16-206). The handling and caring of animals conformed to the guidelines established in order to comply with the current international laws and policies (NIH Guide for the Care and Use of Laboratory Animals, NIH Publication No. 85-23, 1985, revised 1996). Every effort was made to reduce the number of animals used and minimize the suffering of animals caused by the procedures used in the present study.

### 2.2. Drug Treatment

**Figure 1:**
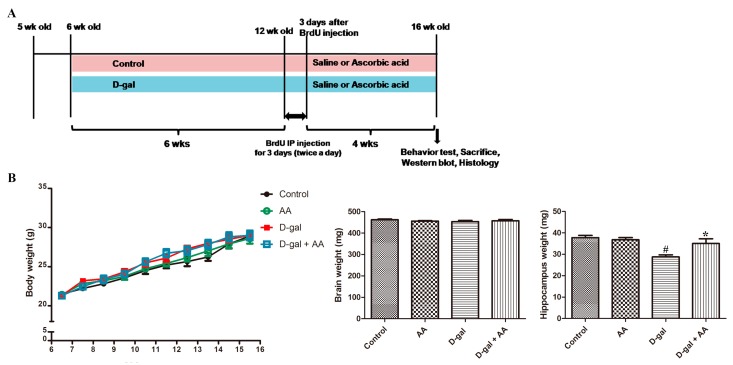
Experimental design (**A**) and changes in body and brain weights (**B**) in control, D-galactose (D-gal), ascorbic acid (150 mg/kg; AA), and ascorbic acid (150 mg/kg)-fed D-gal (D-gal + AA) groups (*n* = 27 per group). 5-bromo-2′-deoxyuridine (BrdU) was intraperitoneally (IP) injected. # *p* < 0.05, indicating a significant difference between control and D-gal groups. * *p* < 0.05, indicating a significant difference between D-gal and D-gal + AA groups. Data are presented as means ± SEM. wk: week.

After one-week acclimation to the condition of housing facilities, the animals were divided into the following four groups: control (CTL), ascorbic acid (AA), D-gal, and ascorbic acid-administered D-gal (D-gal-AA) groups, as described in Figure 1. Mice aged six weeks were subcutaneously administered D-gal (150 mg/kg/day) once a day for 10 weeks [17]. After six weeks of D-gal treatment, the animals were co-administered physiological saline or ascorbic acid (150 mg/kg/day) with oral gavage daily for four weeks (Figure 1). Studies have demonstrated that the dose of ascorbic acid (150 mg/kg/day) adopted in the present study has a favorable effectiveness in mice and rats [18,19,20].

### 2.3. Body Weight

Body weight was measured every Monday morning during the experiment until the end of the experiment.

### 2.4. 5-bromo-2′-Deoxyuridine Administration

At 12 weeks of age, the animals (*n* = 10 in each group) received an intraperitoneal injection of 5-bromo-2′-deoxyuridine (BrdU; Sigma, St. Louis, MO, USA) at a dosage of 50 mg/kg in saline twice daily for three consecutive days to examine the effects of D-gal and ascorbic acid treatment on the differentiation of BrdU-positive cells into mature neurons in the dentate gyrus [21]. Animals were sacrificed four weeks after the final day of BrdU treatment for histology analysis (Figure 1).

### 2.5. Tissue Processing

Mice in the CTL, AA, D-gal, and D-AA groups (*n* = 10 in each group with BrdU injection) were anesthetized with 1.5 g/kg urethane (Sigma-Aldrich) and were then perfused transcardially with 0.1 M phosphate-buffered saline (PBS, pH 7.4), followed by 4% paraformaldehyde in 0.1 M phosphate buffer (pH 7.4). The brains were removed and post-fixed in the same fixative for 24 h. The brain tissues were cryoprotected by infiltration with 30% sucrose for 48 h. Following equilibration in 30% sucrose in PBS, the brains were serially cut on a cryostat (Leica, Wetzlar, Germany) into 30-μm-thick coronal sections. Subsequently, the sections were collected into 12-well plates containing PBS and were stored in storage solution until further processing.

### 2.6. Immunohistochemistry

In order to obtain accurate data, immunohistochemistry was carefully conducted under the same conditions. Five tissue sections were selected at 180 μm apart between 1.46 and 2.46 mm posterior to the bregma, according to a mouse atlas [22]. The sections were sequentially treated with 0.3% hydrogen peroxide (H_2_O_2_) in 0.1 M PBS and 10% normal horse serum in 0.1 M PBS. Subsequently, the sections were incubated with diluted rabbit anti-Ki67 antibodies (1:500; Abcam, Cambridge, UK) or goat anti-doublecortin (DCX) antibodies (1:50; Santa Cruz Biotechnology Inc., Santa Cruz, CA, USA) overnight, and they were then exposed to biotinylated goat anti-rabbit or rabbit anti-goat IgG (1:400; Vector Labs., Burlingame, CA, USA) and streptavidin-peroxidase complex (1:400; Vector Labs., Burlingame, CA, USA). The sections were visualized by a reaction with 3,3’-diaminobenzidine tetrahydrochloride (Sigma, St. Louis, MO, USA).

### 2.7. Double Immunofluorescence

Double immunofluorescence staining was performed as described in the previous study [21]. DNA denaturation was conducted for BrdU immunostaining. Briefly, five sections per animal were incubated in 2 N HCl for DNA hydrolysis and then in boric acid for neutralization, and thereafter, the sections were incubated in a mixture of rat anti-BrdU antibody (1:200; BioSource International, Camarillo, CA, USA) and mouse anti-neuronal nuclear protein (NeuN) antibody (1:500; Millipore, Billerica, MA, USA) for 24 h at 4 °C. After washing five times for 7 min each with 0.01 M PBS, the sections were then incubated in a mixture of FITC-conjugated anti-rat IgG (1:200; Vector Labs., Burlingame, CA, USA) and Texas Red-conjugated anti-mouse IgG (1:500; Vector Labs., Burlingame, CA, USA) for 2 h at room temperature. Immunofluorescence was observed using an Olympus BX51 microscope (Olympus, Tokyo, Japan) equipped with a digital camera (DP71, Olympus, Tokyo, Japan) connected to a personal computer monitor.

### 2.8. Quantification of Histology

To elucidate the effects of D-gal and ascorbic acid on cell proliferation, neuroblast differentiation, and neuronal integration, the regions of interest in the dentate gyrus were analyzed using an image analysis system. Images were calibrated into an array of 512 × 512 pixels corresponding to a complete dentate gyrus (primary magnification, 100 ×). Pixel resolution was at 256 gray levels. The numbers of Ki67-, DCX-, BrdU-, or BrdU/NeuN-immunoreactive cells in the dentate gyrus in the hippocampus were counted using an image analysis system equipped with a computer-based CCD camera (Optimas 6.5 software, CyberMetrics, Scottsdale, AZ, USA). The cell counts obtained from all sections of each mouse brain were averaged.

### 2.9. Western Blot Analysis

We conducted immunoblotting analysis to examine the effects of D-gal on the protein expression levels of DCX, synaptophysin, phosphorylated Ca^2+^/calmodulin-dependent protein kinase (pCAMK) II, brain-derived neurotrophic factor (BDNF), sirtuin1 (Sirt1), caveolin-1, superoxide dismutase 1 (SOD1), SOD2, interleukin 1 beta (IL1β), and tumor necrosis factor alpha (TNFα) in the hippocampus in CTL, D-gal, and D-AA mice. A total of seven animals in each group were sacrificed by decapitation after anesthesia with urethane (2 g/kg). The brains were removed and weighed, and the hippocampi were then dissected with a surgical blade and were also weighed. The whole hippocampi were stored at −80 °C until further use. The tissues were homogenized in 50 mM PBS (pH 7.4) containing 0.1 mM ethylene glycol bis (2-aminoethyl ether)-N,N,N′,N′ tetraacetic acid (pH 8.0), 0.2% nonidet P-40, 10 mM ethylendiamine tetraacetic acid (pH 8.0), 15 mM sodium pyrophosphate, 100 mM β-glycerophosphate, 50 mM sodium fluoride, 150 mM sodium chloride, 2 mM sodium orthovanadate, 1 mM phenylmethylsulfonyl fluoride, and 1 mM dithiothreitol (DTT). After centrifugation, the supernatants were collected, and the protein levels were determined using a Micro Bicinchoninic acid (BCA) protein assay kit with bovine serum albumin as the standard (Pierce Chemical, Rockford, IL, USA). Aliquots containing 40 μg of total protein were boiled in loading buffer containing 150 mM Tris (pH 6.8), 3 mM DTT, 6% sodium dodecyl sulfate, 0.3% bromophenol blue, and 30% glycerol. The aliquots were then loaded onto a polyacrylamide gel. After gel electrophoresis, the separated proteins were transferred to a polyvinylidene difluoride (PVDF) membrane (BIO-RAD). To reduce background staining, we incubated PVDF membranes with 5% non-fat dry milk in Tris-buffered saline with 1% Tween-20 (TBST) for 1 h at room temperature. Subsequently, the PVDF membranes were incubated with mouse anti-DCX (1:500; Santa Cruz Biotechnology, Santa Cruz, CA, USA), mouse anti-Sirt1 (1:1000; Santa Cruz Biotechnology), rabbit anti-synaptophysin (1:5,000; Abcam, Cambridge, UK), rabbit anti-caveolin-1 (1:1,500; Santa Cruz Biotechnology), rabbit anti-SOD1 (1:1000; Santa Cruz Biotechnology), goat anti-SOD2 (1:1000; Santa Cruz Biotechnology), rabbit anti-BDNF (1:1000; Novus, Littleton, CO, USA), rabbit anti-phosphorylated CAMKII (1:1000; Abcam), CAMKII (rabbit, 1:500, GeneTex, Irvine, CA, USA), rabbit anti-IL1β (1:1000; GeneTex, Irvine, CA, USA), or rabbit anti-TNFα (1:2000; Abcam, Cambridge, UK) antibodies overnight at 4 °C. The PVDF membranes were washed three times (10 min each in TBST); incubated with horseradish peroxidase-conjugated anti-mouse IgG, anti-goat IgG, or anti-rabbit IgG; and then visualized with an enhanced luminol-based chemiluminescent kit (Pierce Chemical, Rockford, IL, USA). The blots were scanned by an electronically cooled CCD camera system (Fujifilm LAS-1000), and the chemifluorescent images were captured. The blots were densitometrically scanned for quantification of the relative optical density of each band using NIH Image 1.59 software. The data were normalized against those of β-actin.

### 2.10. Object Location Task

An object location task was performed as previously described, with minor modifications [17]. The experimental apparatus was a rectangular open field box made of white Plexiglass (40 × 40 × 40 cm) with distinct visual cues in one of the walls. Before training, each mouse (*n* = 10 in each group) was habituated to the experimental apparatus for 10 min a day for two consecutive days in the absence of objects. During the training phase, mice were placed in the experimental apparatus with two identical objects and were allowed to explore for 10 min. During the test phase, mice were re-exposed to the experimental apparatus for 10 min, with one of the two objects moved into a novel location. Exploration of each object was scored when the mouse’s nose was touching the object or when its head was oriented toward the object within a distance of 1 cm. To measure the preference for each location, we calculated the discrimination ratio as follows: (time exploring the novel location − time exploring the familiar location)/(time exploring the novel + familiar location).

### 2.11. Statistical Analyses

The data are presented as the mean ± standard error of means. Differences among the means were statistically analyzed by one-way analysis of variance followed by Bonferroni’s post-hoc tests using GraphPad Prism 5.01 software (GraphPad Software, Inc., La Jolla CA, USA). A *p*-value of <0.05 was considered statistically significant.

## 3. Results

### 3.1. Effects of Oral Administration of Ascorbic Acid on Body and Brain Weights in D-gal-Treated Mice

Body and brain weights of the mice in the control, AA, D-gal, and D-gal-AA groups were not significantly different. However, the hippocampus weight was reduced in D-gal-induced brain senescent mice compared with control mice, while ascorbic acid treatment prevented the weight loss of the hippocampus in the D-gal-AA group (Figure 1).

### 3.2. Effects of Oral Administration of Ascorbic Acid on Cell Proliferation in D-gal-Treated Mice

**Figure 2:**
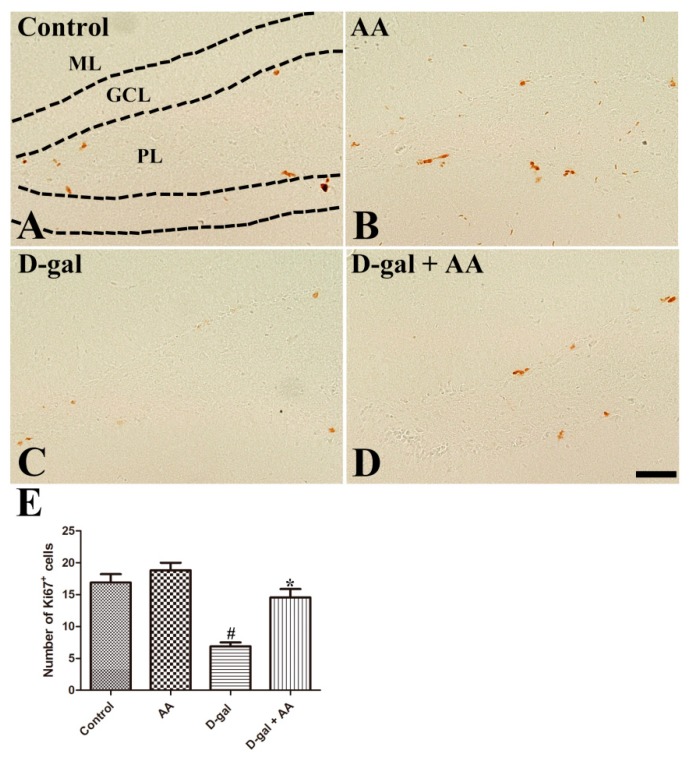
Immunohistochemistry for Ki67 in the dentate gyrus in control (**A**), ascorbic acid (AA) (**B**), D-galactose (D-gal) (**C**), and ascorbic acid (150 mg/kg)-fed D-gal (**D**) groups. Ki67-positive nuclei are observed in the subgranular zone of the dentate gyrus (arrows). Note that numbers of Ki67-positive nuclei significantly decrease in the D-gal group, and ascorbic acid treatment significantly increases cell proliferation in the D-gal + AA group. GCL, granule cell layer; ML, molecular layer; PL, polymorphic layer. Scale bar = 50 μm. (**E**) The number of Ki67-positive nuclei in the subgranular zone of the dentate gyrus (*n* = 10 per group; # *p* < 0.05, indicating a significant difference between control and D-gal groups; * *p* < 0.05, indicating a significant difference between D-gal and D-gal + AA groups). Data are presented as means ± SEM.

As shown in Figure 2A–D, Ki67-immunoreactive nuclei of proliferating cells are mainly detected in the subgranular zone of the dentate gyrus. Ascorbic acid treatment did not change the number of Ki67-immunoreactive nuclei in the hippocampus in the AA group compared with the control group. However, the number of Ki67-immunoreactive nuclei was markedly reduced in the D-gal alone group. Notably, ascorbic acid administration significantly increased the number of Ki67-immunoreactive proliferating cells in the D-gal-AA group compared with the D-gal alone group (Figure 2).

### 3.3. Effects of Oral Administration of Ascorbic Acid on Neuroblast Differentiation in D-gal-Treated Mice

**Figure 3:**
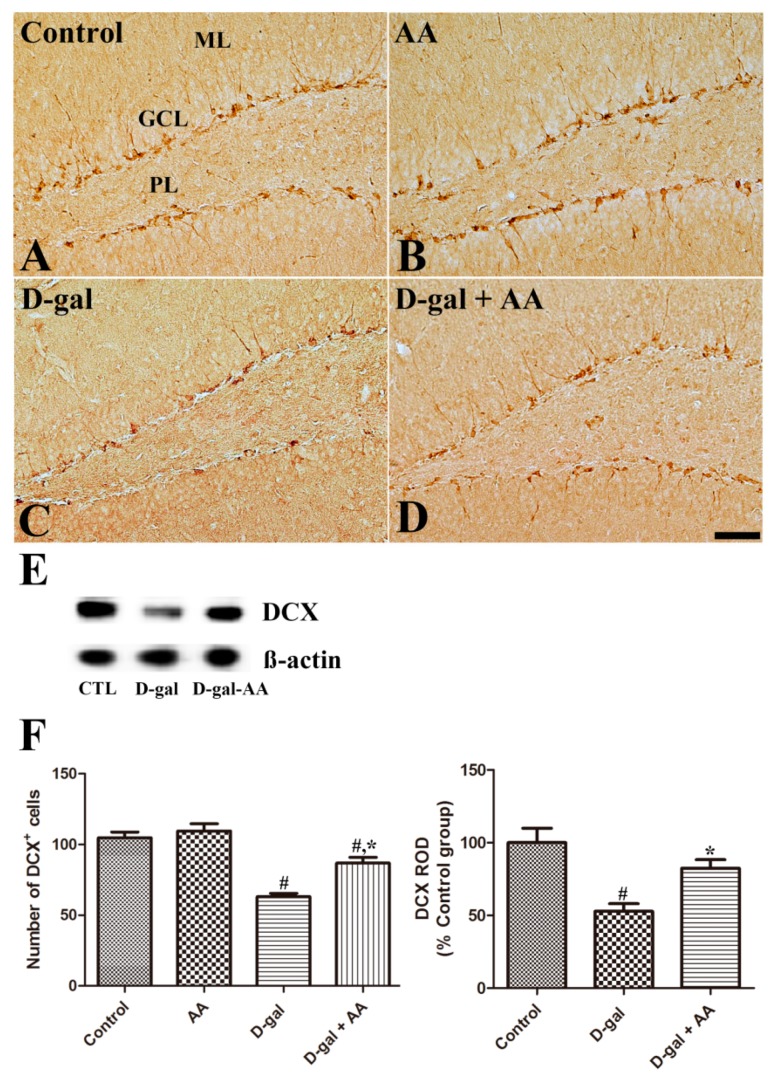
Immunohistochemistry for doublecortin (DCX) in the dentate gyrus in control (CTL) (**A**), ascorbic acid (AA) (**B**), D-galactose (D-gal) (**C**), and ascorbic acid (150 mg/kg)-fed D-gal (**D**) groups. Cell bodies of DCX-positive neuroblasts are located in the subgranular zone of the dentate gyrus, and their dendrites extend in the ML of the dentate gyrus. The number of DCX-positive neuroblasts decreases, and the dendritic complexity is impaired in the D-gal group compared with the control group. Note that ascorbic acid administration significantly increases the number of DCX-positive neuroblasts, and DCX-positive neuroblasts show well-developed dendrites in the D-gal + AA group compared with the D-gal group. GCL, granule cell layer; ML, molecular layer; PL, polymorphic layer. Scale bar = 50 μm. (**E**) Western blot analysis of DCX in the hippocampus in control, D-gal, and D-gal + AA groups. (**F**) The number of DCX-positive neuroblasts in the dentate gyrus. The relative optical density (ROD) of immunoblot bands is expressed as a percentage of the value of the control group (*n* = 10 per group for immunostaining, *n* = 7 per group for immunoblotting; # *p* < 0.05, indicating a significant difference compared with the control group; * *p* < 0.05, indicating a significant difference compared with the D-gal group). Data are presented as means ± SEM.

As shown in Figure 3, cell bodies of DCX-immunoreactive neuroblasts and immature neurons are observed in the subgranular zone, with well-developed dendrites extending into the molecular layer of the dentate gyrus in the control mice. The number of DCX-immunoreactive neuroblasts was not changed in the AA group compared with the control group. However, the numbers and dendritic arbor complexity of DCX-immunoreactive neuroblasts and immature neurons were significantly reduced in the D-gal alone group (Figure 3C,F); notably, the reduction was attenuated by four weeks of ascorbic acid treatment in the D-gal-AA group (Figure 3D,F). The findings by western blotting analysis are in line with those by histological staining. The protein expression levels of DCX significantly decreased in the hippocampus in the D-gal alone group, but the decrease was attenuated by ascorbic acid treatment in the D-gal-AA group (Figure 3E,F).

### 3.4. Effects of Oral Administration of Ascorbic Acid on Neuronal Maturation in D-gal-Treated Mice

**Figure 4:**
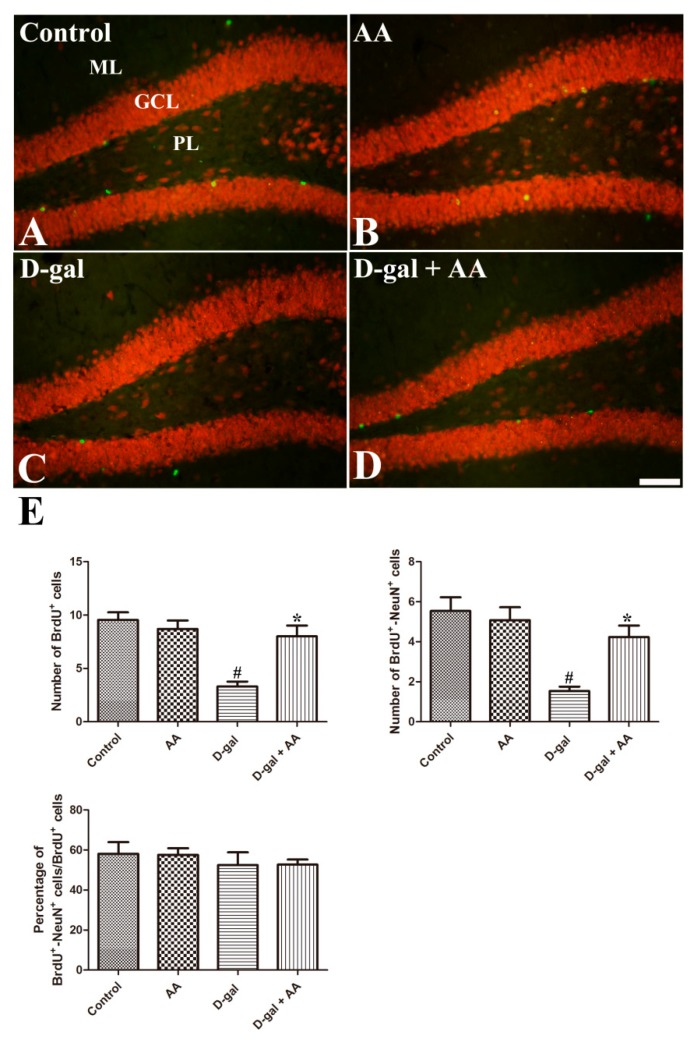
Double immunofluorescence staining of BrdU (green) and NeuN (red) in the dentate gyrus in control (**A**), ascorbic acid (AA) (**B**), D-galactose (D-gal) (**C**), and ascorbic acid (150 mg/kg)-fed D-gal (**D**) groups. Note that D-gal-induced reduction in the number of BrdU-incorporated cells (BrdU^+^) is significantly attenuated by ascorbic acid administration in the D-gal + AA group. The changes in the number of BrdU^+^-NeuN^+^ cells show the same pattern as those of BrdU^+^ cells. GCL, granule cell layer; ML, molecular layer; PL, polymorphic layer. Scale bar = 50 μm. (**E**): The numbers of the BrdU^+^ and BrdU^+^-NeuN^+^ cells in the dentate gyrus of the hippocampus. The percentage of BrdU^+^-NeuN^+^ cells out of the total number of BrdU^+^ cells did not change (*n* = 10 per group; # *p* < 0.05, indicating a significant difference between control and D-gal groups; * *p* < 0.05, indicating a significant difference between D-gal and D-gal + AA groups). Data are presented as means ± SEM.

To determine whether altered cell proliferation and neuronal differentiation further lead to neuronal maturation, we stained the BrdU-incorporated cells with NeuN after four weeks of the last BrdU injection. NeuN is the nuclear marker of mature neurons. As shown in Figure 4A–D, BrdU-positive cells are detected in the subgranular zone and granule cell layer of the dentate gyrus. Chronic treatment of D-gal reduced the numbers of BrdU-incorporated cells and BrdU-NeuN double positive cells in the hippocampus in the D-gal group. BrdU-NeuN double positive cells are newly matured neurons. Ascorbic acid treatment attenuated D-gal-induced reduction of these cells in the hippocampus in the D-gal-AA group (Figure 4). The results suggest that ascorbic acid increases the survival of BrdU-incorporated cells and their neuronal maturation.

### 3.5. Effects of Oral Administration of Ascorbic Acid on SOD1 and SOD2 Expression in D-gal-Treated Mice

**Figure 5:**
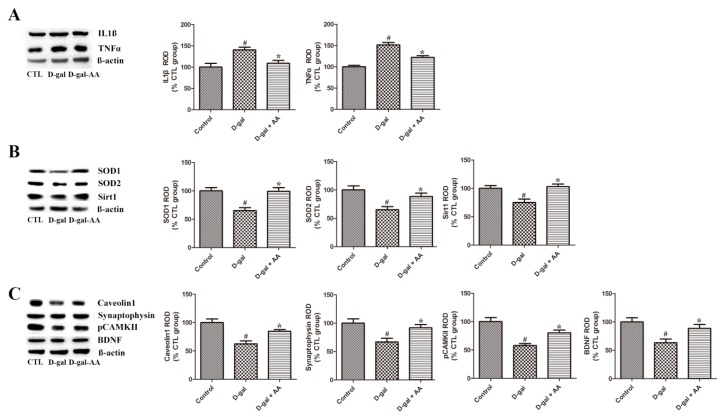
Western blot analysis of interleukin 1 beta (IL1β) and tumor necrosis factor alpha (TNFα) (**A**), superoxide dismutase 1 (SOD1), SOD2, and sirtuin 1 (Sirt1) (**B**), caveolin-1, synaptophysin, phosphorylated Ca^2+^/calmodulin-dependent protein kinase (pCAMK) II, and brain-derived neurotrophic factor (BDNF) (**C**) in the hippocampus in control (CTL), D-galactose (D-gal), and ascorbic acid (150 mg/kg; AA)-fed D-gal groups. Relative optical density (ROD) of immunoblot bands is defined as a percentage of the value of the control group (*n* = 7 per group; # *p* < 0.05, indicating a significant difference between control and D-gal groups; * *p* < 0.05, indicating a significant difference between D-gal and D-gal + AA groups). Data are presented as means ± SEM.

Chronic high levels of D-gal treatment induce the accumulation of oxidative stresses. Fukui et al. (2001) demonstrated that oxidative stress-induced brain damage contributes to learning and memory deficits [23]. We examined the expression levels of SOD1 and SOD2 in the hippocampus to elucidate D-gal-induced oxidative stress and ascorbic acid-mediated antioxidant effects. As we anticipated, long-term D-gal treatment significantly reduced the expression levels of SOD1 and SOD2 in the hippocampus in the D-gal alone group. However, ascorbic acid treatment attenuated D-gal-induced reduction in the two antioxidant enzymes in the hippocampus in the D-gal-AA group (Figure 5). In addition, we also examined whether D-gal treatment affects the glutathione concentration in hippocampal neural progenitor cells (NPC). The cell culture and measurement of total glutathione in hippocampal NPC were conducted according to a previous study (see Supplementary Materials). As shown in Supplementary Figure S1, D-gal treatment on cells reduced the total glutathione concentration, but co-treatment of ascorbic acid with D-gal restored the D-gal-induced glutathione decrease, indicating that D-gal also produces reactive oxygen species in hippocampal neural progenitor cells and that ascorbic acid contributes to the reduction of cellular oxidative stresses induced by D-gal.

### 3.6. Effects of Oral Administration of Ascorbic Acid on IL1β and TNFα Expression in D-gal-Treated Mice

We investigated the effects of D-gal and ascorbic acid treatment on the expression levels of inflammatory mediators (IL-1β and TNFα) in the hippocampus. Both IL-1β and TNFα were significantly upregulated in the hippocampus in the D-gal alone group compared with the control group. However, ascorbic acid co-administration with D-gal abrogated D-gal-induced upregulation of inflammatory cytokines in the hippocampus (Figure 5). These results suggest that long-term D-gal treatment induces neuroinflammation in the hippocampus, while ascorbic acid has anti-inflammatory effects.

### 3.7. Effects of Oral Administration of Ascorbic Acid on Sirt1 and Caveolin-1 Expression in D-gal-Treated Mice

Sirt1 and caveolin-1 are aging-related markers (or indicators). Sirt is a highly conserved family of nicotinamide adenine dinucleotide^+^-dependent deacetylase and class III histone deacetylase (HDAC). HDAC-mediated removal of acetyl groups from acetylated lysine residues of histone leads to chromatin compaction and transcription repression. Sirt1 is an important longevity factor because of its anti-aging action. Caveolin-1, the major structural protein of caveolae, is involved in organization of the synaptic development and signaling. Accelerated aging has been demonstrated in a caveolin-1-knockout model [24]. The expression levels of both Sirt1 and caveolin-1 were significantly reduced in the hippocampus in the D-gal group compared with the control group. However, ascorbic acid treatment attenuated D-gal-induced reduction of Sirt1 and caveolin-1 in the hippocampus in the D-gal-AA group (Figure 5).

### 3.8. Effects of Oral Administration of Ascorbic Acid on Synaptophysin and pCAMK II Expression in D-gal-Treated Mice

Additionally, to elucidate whether altered neurogenesis is linked to changed synaptic plasticity, we investigated related marker proteins in this study. The expression level of synaptophysin, a synaptic maker protein, was reduced by D-gal treatment, and ascorbic acid co-administration attenuated D-gal-induced reduction of synaptophysin. The expression pattern of the activated phosphorylated form of CAMK II (pCAMK II) was also investigated in the hippocampus. CAMK II is essential in the induction of long-term potentiation (LTP) and memory consolidation [25]. Similar to the findings in synaptophysin, D-gal-induced reduction and ascorbic acid-mediated amelioration of the reduction in pCAMK II were detected in the hippocampus (Figure 5). However, the expression level of total CAMKII was not changed by D-gal treatment alone and co-treatment of ascorbic acid with D-gal also did not affect its expression level in the hippocampus (Figure S2).

### 3.9. Effects of Oral Administration of Ascorbic Acid on BDNF Expression in D-gal-Treated Mice

The expression of BDNF was investigated to illustrate the mechanism of the changed neurogenesis and synaptic plasticity. BDNF is an important mediator of structural plasticity, such as adult neurogenesis, synaptogenesis, and functional synaptic plasticity [26]. D-gal treatment significantly reduced the protein expression level of BDNF, while ascorbic acid co-administration attenuated the reduction in the hippocampus in the D-AA group (Figure 5).

### 3.10. Effects of Oral Administration of Ascorbic Acid on Phosphorylated cAMP Response Element-Binding Protein (pCREB) Expression in D-gal-Treated Mice

It is known that the expression of BDNF in the hippocampus is closely regulated by the transcriptional factor of pCREB [27]. We next investigated the effects of D-gal and co-treatment of D-gal with ascorbic acid on pCREB expression in the hippocampus. In all the groups, pCREB-immunoreactive cells were observed in the subgranular zone of the dentate gyrus in the hippocampus (Figure S3). Compared with the control group, the average number of pCREB-immunoreactive cells significantly reduced in the D-gal group. Co-administration of ascorbic acid with D-gal prominently increased the number of pCREB-immunoreactive cells compared with that in the D-gal group. This result suggests that ascorbic acid treatment restored D-gal-induced hippocampal reduction in pCREB-immunoreactive cells and that the restoration of pCREB expression by ascorbic acid is coupled to an increase of BDNF expression.

### 3.11. Effects of Oral Administration of Ascorbic Acid on Spatial Memory in D-gal-Treated Mice

**Figure 6:**
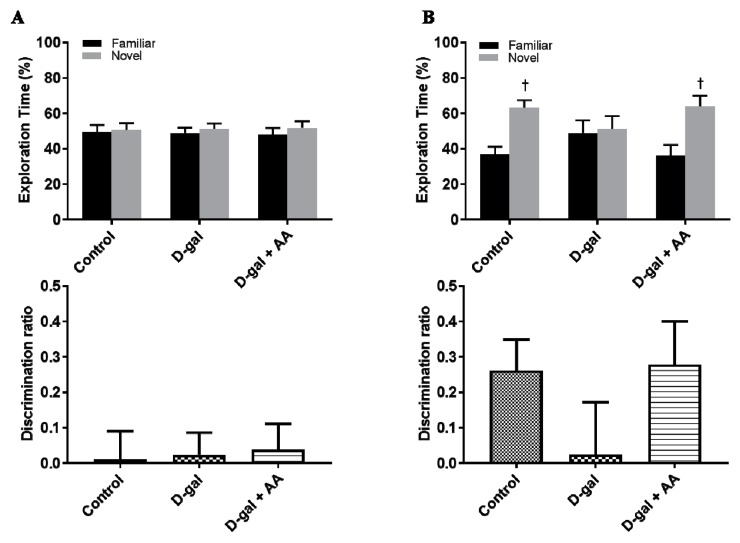
Ascorbic acid (AA) treatment ameliorates object location memory impairment in D-galactose (D-gal)-treated mice. (**A**) During the training phase, mice in all the groups showed no preference for two identical objects. No significant differences were found in the discrimination ratio among these three groups. (**B**) In the test phase, one of the two identical objects was moved into a novel location. Mice in the control and D-gal + AA groups showed a preference for the novel location over the familiar location, whereas mice in the D-gal group did not show preference between the novel location and the familiar location. The discrimination ratio in the D-gal + AA group was significantly higher than that in the D-gal group (*n* = 10 per groups; † *p* < 0.05, indicating a significant difference between familiar and novel location).

Furthermore, to examine whether ascorbic acid treatment leads to functional improvement of the hippocampus, we conducted the object location task, which is used to assess hippocampal-dependent memory. During the training phase, no significant differences were observed among the three groups. However, during the test phase, saline-injected control mice showed a preference for the novel location, while D-gal-treated mice showed no preference for the novel location over the familiar location. Furthermore, a significant decrease in the discrimination ratio was observed in the D-gal alone group compared with the control group. However, ascorbic acid-treated D-gal mice exhibited a preference for the novel location and had a remarkably improved discrimination ratio. These results suggest that ascorbic acid administration reverses D-gal-induced hippocampus-dependent memory impairment (Figure 6).

## 4. Discussion

Previous studies have demonstrated that long-term D-gal treatment induces brain aging [2,4,7,9,17]. Accumulation of oxidative stress is one of the important mechanisms of brain aging [1]. Especially, long-term systemic treatment of high levels of D-gal induces the generation of reactive oxygen species and accelerates brain aging. Ascorbic acid is an essential element for the development of the brain and homeostasis of brain activity [14]. However, the effect of ascorbic acid supplementation on adult brain aging has not been thoroughly investigated. Thus, in the present study, we examined the effect of ascorbic acid in the hippocampus in a D-gal-induced brain aging model.

First, we evaluated the effect of the administration of D-gal and oral gavage of ascorbic acid/D-gal on the body weight of mice. It was found that body weight was not significantly affected during the experiment. Brain weight was not significantly different among the experimental groups, but the hippocampal weight was significantly reduced in the D-gal group; notably, the reduction in hippocampal weight was attenuated by ascorbic acid co-treatment (Figure 1B).

Previous studies, including ours, found that chronic D-gal treatment effectively impaired adult hippocampal neurogenesis [9,17,28]; therefore, we next investigated the effect of D-gal and ascorbic acid co-treatment on adult hippocampal neurogenesis. Although ascorbic acid treatment alone did not affect neurogenesis in the dentate gyrus in the hippocampus, ascorbic acid co-treatment with D-gal attenuated D-gal-induced reduction of Ki67-immunoreactive proliferating cells, DCX-immunoreactive neuroblasts and immature neurons, and BrdU-incorporated NeuN-immunoreactive mature neurons (Figure 2, Figure 3 and Figure 4). The present results are consistent with those of a previous study with an ischemic animal model, in which ascorbic acid co-administration with fluoxetine increased the number of DCX-immunoreactive neuroblasts and improved subsequent functional recovery [29]. Ascorbic acid deficiency causes impairment in hippocampal neurogenesis [13], and ascorbic acid treatment is effective in promoting the proliferation of stem cells [30]. Shin et al. (2004) demonstrated that ascorbic acid promotes the differentiation of embryonic stem cells to neurons by increasing the expression of genes of cell adhesion and development [31]. Thus, ascorbic acid might help mitigate brain aging-related hippocampal dysfunctions.

Previous studies suggested that ascorbic acid-mediated enhancement in structural plasticity is associated with increased neuronal differentiation and antioxidation [31,32]. In this study, we further investigated the effect of ascorbic acid administration on D-gal-induced oxidative stress, and our findings confirm that ascorbic acid restores D-gal-induced reduction of antioxidant enzymes (SOD1 and SOD2) in the hippocampus. Therefore, the reduction of antioxidant enzymes may be responsible for D-gal-induced increased oxidative stresses, and ascorbic acid supplementation attenuates increased oxidative stress. Additionally, Chang et al. (2012) previously demonstrated that the supplementation of ascorbic acid is effective in alleviating lead-induced oxidative stress by increasing the expression levels of both SOD1 and SOD2 in the developing hippocampus [33]. Moreover, other studies indicated that ascorbic acid protects against oxidative stress caused by SOD depletion, chronic stress, and aluminum exposure [20,34,35]. Besides oxidative stress, inflammation is also an important aging-inducing mechanism of D-gal treatment [36]. D-gal treatment promotes the generation of reactive oxygen species that activate inflammatory pathways [37]. Our present study showed that long-term D-gal administration upregulated the protein levels of pro-inflammatory IL-1β and TNFα in the hippocampus, while ascorbic acid co-treatment with D-gal attenuated the upregulation of these inflammatory markers (Figure 5).

After confirming aging phenotypes in the hippocampus, we next sought to identify a critical molecular indicator of brain aging, and we selected Sirt1 and caveolin-1 because they are commonly thought to be associated with brain aging and neurodegeneration [24,38,39,40]. Previous studies demonstrated that Sirt1 is a vital longevity factor because of its anti-aging and anti-oxidative effects [41,42,43,44]. In the present study, D-gal alone-induced brain aging was linked to reduced Sirt1 expression in the hippocampus, while ascorbic acid co-treatment attenuated the decrease of Sirt1. The findings in the present study are similar to those in a previous study [45]. Sirt1 activation induced by resveratrol and melatonin is neuroprotective through antioxidant and anti-inflammatory effects [41,44]. Therefore, it is likely that Sirt1 induction is involved in the beneficial effects of ascorbic acid in the present study [43]. Sirt1 promotes neurogenesis, dendritic/axonal growth, and synaptic plasticity [39]. Recent studies indicate that the caveolin-1 knockdown or knockout condition accelerates senescence by inactivating Sirt1 [24,40]. In addition, the expression level of caveolin-1 is significantly reduced in the hippocampus in 24-month-old aged rats [46]. Collectively, ascorbic acid-mediated induction of caveolin-1 may be associated with the restoration of neurogenesis because ascorbic acid is involved in BDNF signaling-related pro-survival and pro-growth [47]. In fact, in the ischemic brain, caveolin-1 is required for neuroprotection against cell injury and death [48].

Additionally, we evaluated the expression levels of a synaptic marker protein (synaptophysin), the activated form of CAMK II (pCAMK II), and BDNF in the hippocampus. Chronic D-gal alone treatment reduced the protein levels of synaptophysin, pCAMK II, and BDNF, while ascorbic acid co-treatment with D-gal restored the protein levels to near their respective control levels. The results in the present study are supported by those in previous studies, which demonstrated D-gal-induced synaptogenesis and LTP impairment in the hippocampus [17,46]. Ascorbic acid is effective in reversing lead-induced impairment of LTP in the hippocampus [49]. As demonstrated by immunoblotting, the expression pattern of Sirt1 (D-gal alone-induced reduction and ascorbic acid-mediated increase) is similar to that of BDNF, suggesting that Sirt1 contributes to the enhancement of synaptic plasticity and memory by upregulating the expression of BDNF [39]. Therefore, our findings indicate that ascorbic acid has anti-aging effects, and both Sirt1 and BDNF might be responsible for the recovery of structural plasticity in the hippocampus.

Moreover, we conducted the novel location recognition test to evaluate the memory function in the D-gal-induced aging model [50]. Similar to naturally aged mice, D-gal-induced aging mice show memory impairment [51]. As demonstrated in a previous study with an Alzheimer’s disease model [20], ascorbic acid co-treatment ameliorates D-gal-induced memory impairment. Noguchi-Shinohara et al. (2018) recently found that a high blood level of ascorbic acid is associated with the amelioration of apolipoprotein E E4-related cognitive decline in elderly women [52]. Because ascorbic acid has a low toxicity and is a low-cost compound, it could be one of the representative antioxidants that are effective in delaying the onset and progression of Alzheimer’s disease in humans [53].

It is worth noting the dose of ascorbic acid used in the present study. We used a relatively high dose of ascorbic acid to observe anti-brain aging against D-galactose. If ascorbic acid is applied for a translational medicine as an anti-brain aging agent based on the present study, the high-dosage of ascorbic acid could be arguable. However, it is unlikely to do harm with this dosage, since it is well-known that ascorbic acid is safe with even a high dose [54]. Alternatively, we can use less ascorbic acid with other anti-oxidants such as glutathione, as shown in previous reports, to enhance the anti-oxidant activity of ascorbic acid [55]. Thus, co-treatment might also contribute to further lowering the burden of D-galactose conversion to D-galacticol by D-galactose reductase that oxidizes reduced nicotinamide adenine dinucleotide phosphate (NADPH) to nicotinamide adenine dinucleotide phosphate (NADP) [5]. In future, further studies will be required for the translational application of ascorbic acid alone or co-treatment with other anti-oxidants against human brain aging.

## 5. Conclusions

In summary, ascorbic acid-mediated beneficial effects on brain aging may be achieved by several mechanisms, including the promotion of neurogenesis and synaptic plasticity; increase in endogenous antioxidants; attenuation of neuroinflammation; and enhanced expression of Sirt1, caveolin-1, and BDNF, in the hippocampus impaired by D-gal. These mechanisms might account for the subsequent functional improvement in hippocampus-dependent memory. Therefore, our findings suggest that ascorbic acid is a useful anti-brain aging agent.

## Supplementary Materials

The following are available online at http://www.mdpi.com/2072-6643/11/1/176/s1, Supplementary Methods, Figure S1: Effects of ascorbic acid (AA) on D-galactose (D-gal)-induced decrease of total glutathione concentration in the hippocampal NPCs. Note that co-treatment of ascorbic acid with D-galactose attenuates D-galactose alone-induced reduction of total glutathione in in vitro cell assay. # *p* < 0.01, indicating a significant difference from control. * *p* < 0.01, indicating a significant difference from D-gal alone groups (*n* = 9), Figure S2: Effects of ascorbic acid (AA) on D-galactose (D-gal)-induced alterations of CAMKII expression level. Western blot analysis of CAMKII was analyzed by comparing the relative optical density (ROD) of immunoblot bands, which are demonstrated as a percentage of the value of control group (*n* = 7 per group; # *p* < 0.05, indicating a significant difference between control and D-gal groups; * *p* < 0.05, indicating a significant difference between D-gal and D-gal + AA groups). Data are presented as means ± SEM, Figure S3: Immunohistochemistry for pCREB in the dentate gyrus of the in the control (A), ascorbic acid (AA) (B), D-galactose (D-gal) (C), and ascorbic acid (150 mg/kg)-fed D-gal (D) groups. pCREB-positive cells in the subgranular zone of dentate gyrus (arrows) are counted. Note that numbers of pCREB-positive cells are significantly decreased in the D-gal group. In the D-gal+AA group, pCREB-positive cells were significantly increased compared to the D-gal group. GCL, granule cell layer; ML, molecular layer; PL, polymorphic layer. Scale bar = 50 μm. E: The number of pCREB-positive cells in the dentate gyrus of hippocampus (*n* = 3 per group; # *p* < 0.05, indicating a significant difference between control and D-gal groups; * *p* < 0.05, indicating a significant difference between D-gal and D-gal + AA groups). Data are presented as means ± SEM.

## References

[1] Mao P., Reddy P.H. (2011). Aging and amyloid beta-induced oxidative DNA damage and mitochondrial dysfunction in Alzheimer’s disease: Implications for early intervention and therapeutics. Biochim. Biophys. Acta.

[2] Feng Y., Yu Y.H., Wang S.T., Ren J., Camer D., Hua Y.Z., Zhang Q., Huang J., Xue D.L., Zhang X.F. (2016). Chlorogenic acid protects D-galactose-induced liver and kidney injury via antioxidation and anti-inflammation effects in mice. Pharm. Biol..

[3] Floyd R.A., Hensley K. (2002). Oxidative stress in brain aging. Implications for therapeutics of neurodegenerative diseases. Neurobiol. Aging.

[4] Wu W., Wang X., Xiang Q., Meng X., Peng Y., Du N., Liu Z., Sun Q., Wang C., Liu X. (2014). Astaxanthin alleviates brain aging in rats by attenuating oxidative stress and increasing BDNF levels. Food Funct..

[5] Song X., Bao M., Li D., Li Y.M. (1999). Advanced glycation in D-galactose induced mouse aging model. Mech. Ageing Dev..

[6] Banji D., Banji O.J., Dasaroju S., Kranthi K.C. (2013). Curcumin and piperine abrogate lipid and protein oxidation induced by D-galactose in rat brain. Brain Res..

[7] Chang Y.M., Chang H.H., Kuo W.W., Lin H.J., Yeh Y.L., Padma Viswanadha V., Tsai C.C., Chen R.J., Chang H.N., Huang C.Y. (2016). Anti-apoptotic and pro-survival effect of alpinate oxyphyllae fructus (AOF) in a d-galactose-induced aging heart. Int. J. Mol. Sci..

[8] Budni J., Garcez M.L., Mina F., Bellettini-Santos T., da Silva S., Luz A.P.D., Schiavo G.L., Batista-Silva H., Scaini G., Streck E.L. (2017). The oral administration of D-galactose induces abnormalities within the mitochondrial respiratory chain in the brain of rats. Metab. Brain Dis..

[9] Nam S.M., Choi J.H., Yoo D.Y., Kim W., Jung H.Y., Kim J.W., Kang S.Y., Park J., Kim D.W., Kim W.J. (2013). Valeriana officinalis extract and its main component, valerenic acid, ameliorate D-galactose-induced reductions in memory, cell proliferation, and neuroblast differentiation by reducing corticosterone levels and lipid peroxidation. Exp. Gerontol..

[10] Lu J., Wu D.M., Hu B., Cheng W., Zheng Y.L., Zhang Z.F., Ye Q., Fan S.H., Shan Q., Wang Y.J. (2010). Chronic administration of troxerutin protects mouse brain against D-galactose-induced impairment of cholinergic system. Neurobiol. Learn. Mem..

[11] Barrita J., Sánchez M., Morales-Gonzalez J.A. (2013). Antioxidant role of ascorbic acid and his protective effects on chronic diseases. Oxidative Stress and Chronic Degenerative Diseases—A Role Antioxidants.

[12] Varvara M., Bozzo G., Celano G., Disanto C., Pagliarone C.N., Celano G.V. (2016). The use of ascorbic acid as a food additive: Technical-legal issues. Ital. J. Food Saf..

[13] Tveden-Nyborg P., Vogt L., Schjoldager J.G., Jeannet N., Hasselholt S., Paidi M.D., Christen S., Lykkesfeldt J. (2012). Maternal vitamin C deficiency during pregnancy persistently impairs hippocampal neurogenesis in offspring of guinea pigs. PLoS ONE.

[14] Oke A.F., May L., Adams R.N. (1987). Ascorbic acid distribution patterns in human brain. A comparison with nonhuman mammalian species. Ann. N. Y. Acad. Sci..

[15] Patnaik B.K., Kanungo M.S. (1966). Ascorbic acid and aging in the rat. Uptake of ascorbic acid by teeth and concentration of various forms of ascorbic acid in different organs. Biochem. J..

[16] Khaw K.T., Bingham S., Welch A., Luben R., Wareham N., Oakes S., Day N. (2001). Relation between plasma ascorbic acid and mortality in men and women in EPIC-Norfolk prospective study: A prospective population study. Lancet.

[17] Nam S.M., Hwang H., Seo M., Chang B.J., Kim H.J., Choi S.H., Rhim H., Kim H.C., Cho I.H., Nah S.Y. (2018). Gintonin attenuates D-galactose-induced hippocampal senescence by improving long-term hippocampal potentiation, neurogenesis, and cognitive functions. Gerontology.

[18] Colomina M.T., Gómez M., Domingo J.L., Corbella J. (1994). Lack of maternal and developmental toxicity in mice given high doses of aluminium hydroxide and ascorbic acid during gestation. Pharmacol. Toxicol..

[19] Nair A.B., Jacob S. (2016). A simple practice guide for dose conversion between animals and human. J. Basic Clin. Pharm..

[20] Olajide O.J., Yawson E.O., Gbadamosi I.T., Arogundade T.T., Lambe E., Obasi K., Lawal I.T., Ibrahim A., Ogunrinola K.Y. (2017). Ascorbic acid ameliorates behavioural deficits and neuropathological alterations in rat model of Alzheimer’s disease. Environ. Toxicol. Pharmacol..

[21] Nam S.M., Kim Y.N., Kim J.W., Kyeong D.S., Lee S.H., Son Y., Shin J.H., Kim J., Yi S.S., Yoon Y.S. (2016). Hairy and enhancer of split 6 (Hes6) deficiency in mouse impairs neuroblast differentiation in dentate gyrus without affecting cell proliferation and integration into mature neurons. Cell. Mol. Neurobiol..

[22] Franklin K.B.J., Paxinos G. (2012). The Mouse Brain in Stereotaxic Coordinates.

[23] Fukui K., Onodera K., Shinkai T., Suzuki S., Urano S. (2001). Impairment of learning and memory in rats caused by oxidative stress and aging, and changes in antioxidative defense systems. Ann. N. Y. Acad. Sci..

[24] Head B.P., Peart J.N., Panneerselvam M., Yokoyama T., Pearn M.L., Niesman I.R., Bonds J.A., Schilling J.M., Miyanohara A., Headrick J. (2010). Loss of caveolin-1 accelerates neurodegeneration and aging. PLoS ONE.

[25] Lisman J., Yasuda R., Raghavachari S. (2012). Mechanisms of CaMKII action in long-term potentiation. Nat. Rev. Neurosci..

[26] Lee J., Duan W., Mattson M.P. (2002). Evidence that brain-derived neurotrophic factor is required for basal neurogenesis and mediates, in part, the enhancement of neurogenesis by dietary restriction in the hippocampus of adult mice. J. Neurochem..

[27] Luo Y., Kuang S., Li H., Ran D., Yang J. (2017). cAMP/PKA-CREB-BDNF signaling pathway in hippocampus mediates cyclooxygenase 2-induced learning/memory deficits of rats subjected to chronic unpredictable mild stress. Oncotarget.

[28] Hong X.P., Chen T., Yin N.N., Han Y.M., Yuan F., Duan Y.J., Shen F., Zhang Y.H., Chen Z.B. (2016). Puerarin ameliorates D-galactose induced enhanced hippocampal neurogenesis and tau hyperphosphorylation in rat brain. J. Alzheimers Dis..

[29] Corbett A.M., Sieber S., Wyatt N., Lizzi J., Flannery T., Sibbit B., Sanghvi S. (2015). Increasing neurogenesis with fluoxetine, simvastatin and ascorbic acid leads to functional recovery in ischemic stroke. Recent Pat. Drug Deliv. Formul..

[30] Cao N., Liu Z., Chen Z., Wang J., Chen T., Zhao X., Ma Y., Qin L., Kang J., Wei B. (2012). Ascorbic acid enhances the cardiac differentiation of induced pluripotent stem cells through promoting the proliferation of cardiac progenitor cells. Cell Res..

[31] Shin D.M., Ahn J.I., Lee K.H., Lee Y.S., Lee Y.S. (2004). Ascorbic acid responsive genes during neuronal differentiation of embryonic stem cells. Neuroreport.

[32] Ochiai Y., Kaburagi S., Obayashi K., Ujiie N., Hashimoto S., Okano Y., Masaki H., Ichihashi M., Sakurai H. (2006). A new lipophilic pro-vitamin C, tetra-isopalmitoyl ascorbic acid (VC-IP), prevents UV-induced skin pigmentation through its anti-oxidative properties. J. Dermatol. Sci..

[33] Chang B.J., Jang B.J., Son T.G., Cho I.H., Quan F.S., Choe N.H., Nahm S.S., Lee J.H. (2012). Ascorbic acid ameliorates oxidative damage induced by maternal low-level lead exposure in the hippocampus of rat pups during gestation and lactation. Food Chem. Toxicol..

[34] Moretti M., Colla A., de Oliveira Balen G., dos Santos D.B., Budni J., de Freitas A.E., Farina M., Severo Rodrigues A.L. (2012). Ascorbic acid treatment, similarly to fluoxetine, reverses depressive-like behavior and brain oxidative damage induced by chronic unpredictable stress. J. Psychiatr. Res..

[35] Tamari Y., Nawata H., Inoue E., Yoshimura A., Yoshii H., Kashino G., Seki M., Enomoto T., Watanabe M., Tano K. (2013). Protective roles of ascorbic acid in oxidative stress induced by depletion of superoxide dismutase in vertebrate cells. Free Radic. Res..

[36] Ali T., Badshah H., Kim T.H., Kim M.O. (2015). Melatonin attenuates D-galactose-induced memory impairment, neuroinflammation and neurodegeneration via RAGE/NF-K B/JNK signaling pathway in aging mouse model. J. Pineal Res..

[37] Rehman S.U., Shah S.A., Ali T., Chung J.I., Kim M.O. (2017). Anthocyanins reversed D-galactose-induced oxidative stress and neuroinflammation mediated cognitive impairment in adult rats. Mol. Neurobiol..

[38] Liu Y., Liang Z., Liu J., Zou W., Li X., Wang Y., An L. (2013). Downregulation of caveolin-1 contributes to the synaptic plasticity deficit in the hippocampus of aged rats. Neural Regen. Res..

[39] Ng F., Wijaya L., Tang B.L. (2015). SIRT1 in the brain-connections with aging-associated disorders and lifespan. Front. Cell. Neurosci..

[40] Yu D.M., Jung S.H., An H.T., Lee S., Hong J., Park J.S., Lee H., Lee H., Bahn M.S., Lee H.C. (2017). Caveolin 1 deficiency induces premature senescence with mitochondrial dysfunction. Aging Cell.

[41] Pallauf K., Rimbach G., Rupp P.M., Chin D., Wolf I.M. (2016). Resveratrol and lifespan in model organisms. Curr. Med. Chem..

[42] Raynes R., Brunquell J., Westerheide S.D. (2013). Stress inducibility of SIRT1 and its role in cytoprotection and cancer. Genes Cancer.

[43] Wei W., Li L., Zhang Y., Yang J., Zhang Y., Xing Y. (2014). Vitamin C protected human retinal pigmented epithelium from oxidant injury depending on regulating SIRT1. Sci. World J..

[44] Zhao L., An R., Yang Y., Yang X., Liu H., Yue L., Li X., Lin Y., Reiter R.J., Qu Y. (2015). Melatonin alleviates brain injury in mice subjected to cecal ligation and puncture via attenuating inflammation, apoptosis, and oxidative stress: The role of SIRT1 signaling. J. Pineal Res..

[45] Kou X., Liu X., Chen X., Li J., Yang X., Fan J., Yang Y., Chen N. (2016). Ampelopsin attenuates brain aging of D-gal-induced rats through miR-34a-mediated SIRT1/mTOR signal pathway. Oncotarget.

[46] Zhan P.Y., Peng C.X., Zhang L.H. (2014). Berberine rescues D-galactose-induced synaptic/memory impairment by regulating the levels of Arc. Pharmacol. Biochem. Behav..

[47] Head B.P., Hu Y., Finley J.C., Saldana M.D., Bonds J.A., Miyanohara A., Niesman I.R., Ali S.S., Murray F., Insel P.A. (2011). Neuron-targeted caveolin-1 protein enhances signaling and promotes arborization of primary neurons. J. Biol. Chem..

[48] Head B.P., Patel H.H., Tsutsumi Y.M., Hu Y., Mejia T., Mora R.C., Insel P.A., Roth D.M., Drummond J.C., Patel P.M. (2008). Caveolin-1 expression is essential for N-methyl-D-aspartate receptor-mediated Src and extracellular signal-regulated kinase 1/2 activation and protection of primary neurons from ischemic cell death. FASEB J..

[49] Karamian R., Komaki A., Salehi I., Tahmasebi L., Komaki H., Shahidi S., Sarihi A. (2015). Vitamin C reverses lead-induced deficits in hippocampal synaptic plasticity in rats. Brain Res. Bull..

[50] Vogel-Ciernia A., Wood M.A. (2014). Examining object location and object recognition memory in mice. Curr. Protoc. Neurosci..

[51] Wimmer M.E., Hernandez P.J., Blackwell J., Abel T. (2012). Aging impairs hippocampus-dependent long-term memory for object location in mice. Neurobiol. Aging.

[52] Noguchi-Shinohara M., Abe C., Yuki-Nozaki S., Dohmoto C., Mori A., Hayashi K., Shibata S., Ikeda Y., Sakai K., Iwasa K. (2018). Higher blood vitamin C levels are associated with reduction of apolipoprotein E E4-related risks of cognitive decline in women: The Nakajima study. J. Alzheimers Dis..

[53] Harrison F.E. (2012). A critical review of vitamin C for the prevention of age related cognitive decline and Alzheimer’s disease. J. Alzheimers Dis..

[54] Ehrhart J., Zeevalk G.D. (2003). Cooperative interaction between ascorbate and glutathione during mitochondrial impairment in mesencephalic cultures. J. Neurochem..

[55] Dunham W.B., Zuckerkandl E., Reynolds R., Willoughby R., Marcuson R., Barth R., Pauling L. (1982). Effects of intake of L-ascorbic acid on the incidence of dermal neoplasms induced in mice by ultraviolet light. Proc. Natl. Acad. Sci. USA.

