# Supplementary material for: Ascorbic Acid Mitigates D-galactose-Induced Brain Aging by Increasing Hippocampal Neurogenesis and Improving Memory Function
Source: Nutrients. 2019 Jan 15;11(1):176. doi: 10.3390/nu11010176 (PMC6356429; doi:10.3390/nu11010176)
Supplement: Supplementary file 1 [file nutrients-11-00176-s001.pdf]

Supplementary data

Supplementary Methods

Tissue processing, paraffin tissue embedding, and immunohistochemistry

For histological studies, the control, ascorbic acid (AA), D-gal alone, and D-gal + ascorbic acid (150 mg/kg) groups (n = 3 in each group) were anesthetized with 1.5 g/kg urethane (Sigma-Aldrich) and perfused transcardially with 0.1 M phosphate-buffered saline (PBS, pH 7.4) followed by 4% paraformaldehyde in 0.1 M phosphate buffer (pH 7.4). The brains were removed and post-fixed in the same fixative for 12 h. Brain tissues were dehydrated with graded concentrations of alcohol and xylene for embedding in paraffin. Three-micrometer-thick sections were serially cut using a microtome (Leica, Wetzlar, Germany). The brain sections were selected between -1.46 mm and -2.46 mm posterior to the bregma in reference to a mouse atlas [1] for each animal. The sections were mounted on silane-coated slides (Muto-glass, Tokyo, Japan). The phosphorylated cAMP response element-binding protein (pCREB) immunohistochemical staining of the paraffin-embedded block was performed according to a previous study [2]. Briefly, for antigen retrieval, the sections were placed in 400-mL jars filled with citrate buffer (pH 6.0) and heated in a microwave oven (three heating cycles of 5 min each). Thereafter, the slides were cooled to room temperature and washed in PBS. The sections were sequentially treated with 0.3% hydrogen peroxide (H_2O_2) in 0.1 M PBS and 10% normal horse serum in 0.1 M PBS. Then, they were incubated with diluted rabbit anti-pCREB (1:1,000; Millipore, Temecula, CA, USA) overnight, and subsequently exposed to biotinylated goat anti-rabbit (diluted 1:400; Vector Labs., Burlingame, CA, USA) and streptavidin peroxidase complex (diluted 1:400, Vector Labs). Following this, the sections were visualized by a reaction with 3,3′-diaminobenzidine tetrahydrochloride (Sigma, St. Louis, MO, USA). The number of pCREB-immunoreactive cells in the subgranular zone in the dentate gyrus of hippocampus was counted using an image analysis system equipped with a computer-based CCD camera (Optimas 6.5 software, CyberMetrics, Scottsdale, AZ, USA). The cell counts from all of the sections of all of the mice were averaged.

Hippocampal neural precursor cell (NPC) culture

Hippocampal NPC cultures were prepared according to the procedure described by Kim et al., (2011) [3]. Briefly, embryos at embryonic day 14.5 (E14.5) were dissected out of C57BL/6 adult pregnant female mice. The hippocampal region of the embryonic brain was isolated in calcium/magnesium free Hanks' Balanced Salt Solution (HBSS). Cells were plated at 2.5 × 104 cells/cm2 on 10-cm-diameter plates coated with 15 µg/ml poly-L-ornithine and 1 µg/ml fibronectin (Invitrogen). Cells were placed in N2 medium supplemented with B27 (Invitrogen) at 37 °C in a 95% air/5% CO2 gas incubator. Basic fibroblast growth factor (bFGF, 20 ng/ml, R&D Systems, Minneapolis, MN, USA) and epidermal growth factor (EGF, 20 ng/ml, R&D Systems) were added daily in order to increase the hippocampal NPC population, and the medium was changed every other day. Cells at 80% confluency were sub-cultured and maintained at 6 × 104 cells/cm2 in B27-supplemented N2 medium containing bFGF and EGF. Differentiation of these sub-cultured cells was induced via the withdrawal of bFGF and EGF, and they were maintained in the differentiation medium (Neurobasal medium supplemented with B27) for three to five days.

Measurement of total glutathione in hippocampal NPC

Hippocampal NPCs were plated in 96-well plates (20,000 cells/well) overnight. Total glutathione levels were measured in hippocampal NPCs using a simple in vitro fluorometric detection assay kit (Abcam ab65322). In brief, cells were divided into three groups; control, D-galactose (300 mM), or ascorbic acid (200 µM) + D-galactose (30 mM), as shown in Figure S2 [2]. The cells were harvested after 24 h. The harvested cells were washed and homogenized by pipetting up and down. After that,

the homogenates were deproteinized and neutralized samples by adding Perchloric acid (PCA) and Potassium Hydroxide (KOH). Measurement of this assay was executed using an ELISA plate reader (SpectraMax® L Microplate Luminometer, Molecular Devices, USA), according to the assay protocol.

Immunoblotting assay

To confirm the effects of D-gal and ascorbic acid on the expression level of total CAMKII in the hippocampus, seven mice in each group were sacrificed by decapitation after urethane (2 g/kg) anesthesia. Mice brains were quickly removed and hippocampus was then dissected with a surgical blade and stored at -80°C for analysis. For immunoblotting, brain tissues were homogenized in 50 mM PBS (pH 7.4) containing 0.1 mM ethyleneglycol bis (2-aminoethyl ether)-N,N,N′,N′ tetraacetic acid (EGTA) (pH 8.0), 0.2% nonidet P-40, 10 mM ethylendiamine tetraacetic acid (EDTA) (pH 8.0), 15 mM sodium pyrophosphate, 100 mM β-glycerophosphate, 50 mM NaF, 150 mM NaCl, 2 mM sodium orthovanadate, 1 mM phenylmethylsulfonyl fluoride (PMSF), and 1 mM dithiothreitol (DTT). After centrifugation, the protein concentration in the supernatants was determined using a Micro BCA protein assay kit with bovine serum albumin as the standard (Pierce Chemical, Rockford, IL, USA). Aliquots containing 80 μg proteins were denatured in loading buffer containing 150 mM Tris (pH 6.8), 3 mM DTT, 6% SDS, 0.3% bromophenol blue, and 30% glycerol. The aliquots were then loaded onto 10% polyacrylamide gel. After electrophoresis, the gels were transferred to nitrocellulose transfer membranes (Pall Corp., East Hills, NY, USA). To reduce background staining, the membranes were incubated with 5% skim milk in Tris-buffered saline (TBS, pH 7.4) containing 0.1% Tween 20 for 1 h, followed by incubation with a primary antibody against CAMKII (rabbit, 1:500, GeneTex, Irvine, CA, USA) overnight at 4°C. The blots were washed three times in TBS containing 0.1% Tween-20 and then incubated with horseradish peroxidase-conjugated anti-rabbit IgG (1:2000). Bands were visualized using SuperSignal® West Pico Chemiluminescent Substrate (Thermo Fisher Scientific, Rockford, IL, USA). The blot was densitometrically scanned for the quantification of relative optical density (ROD) of each band using NIH Image 1.59 software. The data were normalized against β-actin.

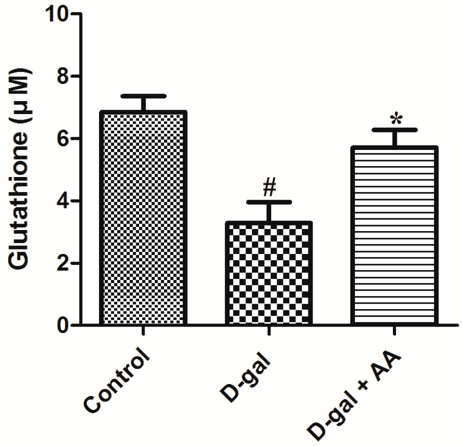

Figure S1. Effects of ascorbic acid (AA) on D-galactose (D-gal)-induced decrease of total glutathione concentration in the hippocampal NPCs. Note that co-treatment of ascorbic acid with D-galactose attenuates D-galactose alone-induced reduction of total glutathione in in vitro cell assay. #P < 0.01, indicating a significant difference from control. *P < 0.01, indicating a significant difference from D-gal alone groups (n = 9).

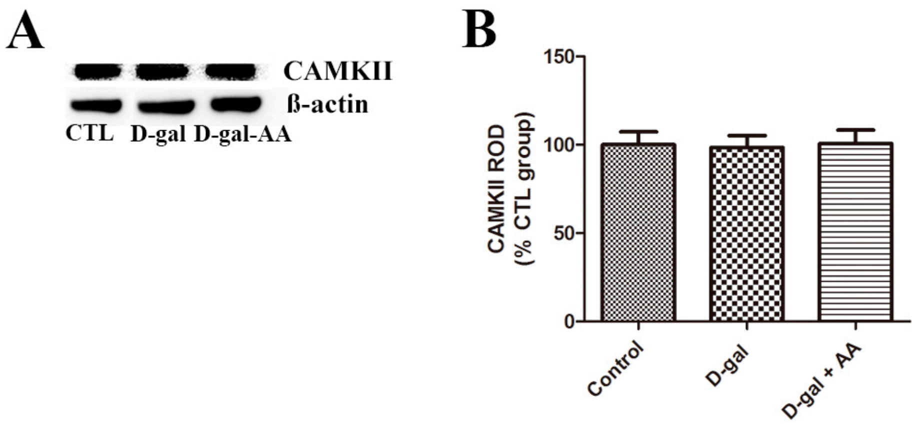

Figure S2. Effects of ascorbic acid (AA) on D-galactose (D-gal)-induced alterations of CAMKII expression level. Western blot analysis of CAMKII was analyzed by comparing the relative optical density (ROD) of immunoblot bands, which are demonstrated as a percentage of the value of control group (n = 7 per group; #P < 0.05, indicating a significant difference between control and D-gal groups; *P < 0.05, indicating a significant difference between D-gal and D-gal + AA groups). Data are presented as means ± SEM.

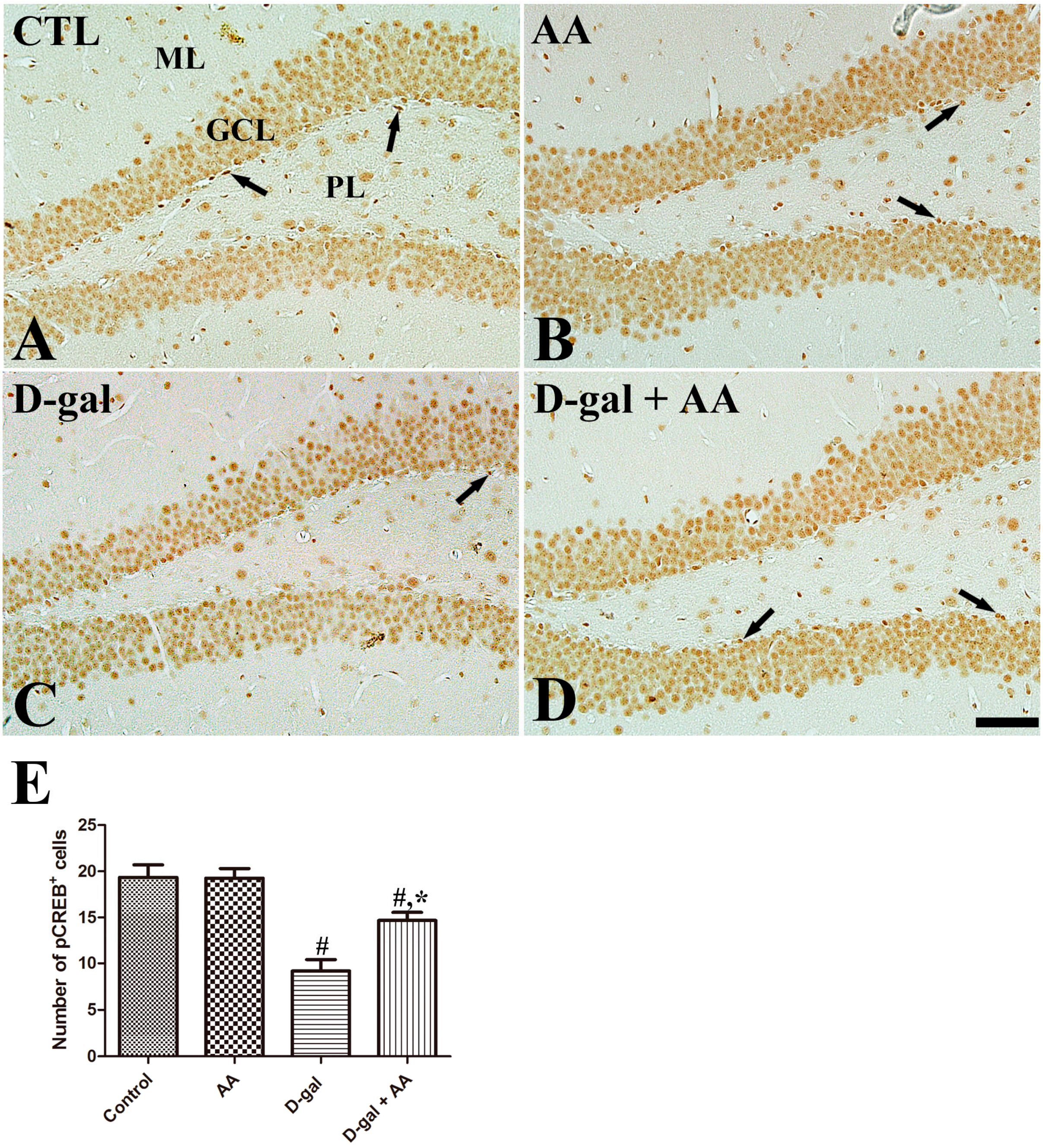

Figure S3. Immunohistochemistry for pCREB in the dentate gyrus of the in the control (A), ascorbic acid (AA) (B), D-galactose (D-gal) (C), and ascorbic acid (150 mg/kg)-fed D-gal (D) groups. pCREB-positive cells in the subgranular zone of dentate gyrus (arrows) are counted. Note that numbers of pCREB-positive cells are significantly decreased in the D-gal group. In the D-gal+AA group, pCREB-positive cells were significantly increased compared to the D-gal group. GCL, granule cell layer; ML, molecular layer; PL, polymorphic layer. Scale bar=50 μm. E: The number of pCREB-positive cells in the dentate gyrus of hippocampus (n = 3 per group; #P < 0.05, indicating a significant difference between control and D-gal groups; *P < 0.05, indicating a significant difference between D-gal and D-gal + AA groups). Data are presented as means ± SEM.

Supplementary References

1. Franklin, K.B.J.; Paxinos, G. *The mouse brain in stereotaxic coordinates*, 4th ed.; Academic Press: San Diego, USA, 2012; ISBN 9780123910578.

2. Nam, S.M.; Hwang, H.; Seo, M.; Chang, B.J.; Kim, H.J.; Choi, S.H.; Rhim, H.; Kim, H.C.; Cho, I.H.; Nah, S.Y. Gintonin attenuates D-galactose-induced hippocampal senescence by improving long-term hippocampal potentiation, neurogenesis, and cognitive functions. *Gerontology* **2018**, *64*, 562-575. doi: 10.1159/000491113.

3. Kim, H.J.; Shin, E.J.; Lee, B.H.; Choi, S.H.; Jung, S.W.; Cho, I.H.; Hwang, S.H.; Kim, J.Y.; Han, J.S.; Chung, C.; Jang, C.G.; Rhim, H.; Kim, H.C.; Nah, S.Y. Oral administration of gintonin attenuates cholinergic impairments by scopolamine, amyloid-β protein, and mouse model of Alzheimer's disease. *Mol. Cells* **2015**,38, 796-805.
